# Three *MYO15A* Mutations Identified in One Chinese Family with Autosomal Recessive Nonsyndromic Hearing Loss

**DOI:** 10.1155/2018/5898025

**Published:** 2018-04-05

**Authors:** Fengguo Zhang, Lei Xu, Yun Xiao, Jianfeng Li, Xiaohui Bai, Haibo Wang

**Affiliations:** ^1^Department of Otorhinolaryngology Head and Neck Surgery, Shandong Provincial Hospital Affiliated to Shandong University, Jinan, China; ^2^Shandong Provincial Key Laboratory of Otology, Jinan, China

## Abstract

Hearing impairment is one of the most common sensory disease, of which more than 50% is attributed to a genetic etiology. The goal of this research is to explore the genetic cause of a Chinese deafness pedigree who was excluded of *GJB2*, *SLC26A4*, or *MtDNA12SrRNA* variants. Three variants, c.3971C>A (p.A1324D), c.4011insA (p.Q1337Qfs^∗^22), and c.9690+1G>A, in the *MYO15A* gene were identified by targeted capture sequencing and Sanger sequencing, and the first two of them were novel. These variants were cosegregated with the disease in this family and absent in 200 normal hearing persons. They were concluded to be pathogenic mutations by phylogenetic analysis and structure modeling. Thus, the combined use of SNPScan assay and targeted capture sequencing is a high-efficiency and cost-effective screening procedure for hereditary hearing loss. Genetic counseling would be important for this family, and our finding would be a great supplement to the mutation spectrum of *MYO15A*.

## 1. Introduction

Hearing impairment is a common sensorineural disease, affecting about one in 500–1000 children, and more than 50% congenital hearing loss is attributable to genetic origins [1]. Approximately, 70% of the deafness without other symptoms is known as nonsyndromic hearing loss (NSHL). Hereditary deafness can be classified as autosomal dominant, autosomal recessive, mitochondrial, and X-linked. Autosomal recessive nonsyndromic hearing loss (ARNSHL) is the most frequent hereditary pattern [2, 3].

Since *GJB2* was identified as the responsible gene for hereditary hearing loss [4], over 80 genes and 2000 mutations have been identified causing NSHL till now (http://deafnessvariationdatabase.org). *MYO15A* gene was revealed to be the disease-causing gene of ARNSHL [5]. Since then, more and more studies on *MYO15A* gene have focused on the mutational analysis. Usually, pathogenic mutations of *MYO15A* gene can cause congenital severe to profound hearing loss in all frequencies [6, 7], whereas some patients are noted to have residual hearings at low frequencies [8].

*MYO15A* gene consisting of 66 exons encodes a protein myosin XVA with 3530 amino acid. Myosin XVA can be classified into two isoforms: class 1 and class 2 [9]. They both contain six same domains: motor, MyTH, IQ, FERM, SH3, and PDZ. In addition, the class 1 isoform also has an N-terminal domain [10–12]. Both two isoforms can be found in the human inner ear [9].

Myosin XVA, expressed at the tips of stereocilia in the cochlea hair cells, is essential for the function of mechanotransduction apparatus [13], which is proved by a myosin XVA-deficient mouse model study [14].

Here, we report a consanguineous Chinese family affected by profound sensorineural hearing loss. We used SNPscan assay and targeted capture sequencing to identify the gene responsible for the deafness in the family. The results identified three compound heterozygous mutations, c.3971C>A (p.A1324D), c.4011insA (p.Q1337Qfs^∗^22), and c.9690+1G>A, in the *MYO15A* gene, and the first two of them were novel. Our finding would be a great supplement for the *MYO15A* pathogenic mutations and would make the genetic counseling available for this family.

## 2. Materials and Methods

### 2.1. Subjects

This work was carried out with the permission of Shandong University ethical committee (number 014). All participants involved in the project signed written informed consent. A Chinese family affected by ARNSHL was recruited. The living family members consisted of four patients (V:3, V:4, IV:4, and IV:5, Figure 1(a)) and eight healthy persons. All affected members showed a bilateral congenital hearing loss. 200 normal persons were collected. All audiometric tests and physical examinations were evaluated at Shandong Provincial Hospital.

### 2.2. Clinical Evaluations

Audiometric assessments consist of auditory brainstem response, pure tone audiometry, distortion product otoacoustic emission, tympanometry, and acoustic stapedial reflex. The classification of deafness was based on the pure tone average (PTA) [15]. Hearing level was sorted into normal hearing (<25 dBHL), mild deafness (>25 and <40 dBHL), moderate deafness (>40 and <60 dBHL), severe deafness (>61 and <80 dBHL), and profound deafness (>80 dBHL) [16].

### 2.3. DNA Samples

A DNA extraction kit (AXYGEN) was employed to extract genomic DNA of all family members and 200 controls. Agarose gel electrophoresis was performed for evaluating the quality and quantity of DNA samples according to the routine protocol,

excluding common deafness genes.

In order to exclude mutations of *GJB2*, *SLC26A4*, and *MtDNA12SrRNA* genes, a “SNPscan assay” was employed. This SNPscan assay from Genesky Biotechnologies Inc. (Shanghai, China) was designed to capture a total of 115 mutations of the three common deafness-causing genes [17, 18]. It was carried out according to the detailed protocol described previously [19].

### 2.4. Targeted Capture Sequencing of Deafness Genes

After excluding common mutations, the targeted deafness gene capture was conducted by BGI Inc. (Wuhan, China). All exons, splicing sites, and flanking intron sequences of 127 hearing loss-related genes (Supplement Material: Table S1) were captured. The workflow was (1) the fragmentization of Genomic DNA into 200–300 base pairs, (2) the construction of the genomic library, (3) the capture of targeted DNA fragments by hybridization to the capture array, (4) sequencing on Illumina HiSeq2000 Analyzers, and (5) data collection and bioinformatics analysis. The SOAPsnp software and the GATK IndelGenotyper were used to identify SNPs and indels, respectively. The NCBI dbSNP, 1000 Genomes, and the in-house database were also involved to filter SNPs [20].

### 2.5. Direct Sanger Sequencing

Using targeted capture sequencing, we identified three candidate mutations. In order to determine whether these variants were cosegregated with hearing loss in the family, the PCR and Sanger sequencing were employed. Therefore, the following primers were synthesized: 5′-GATTGCCTGGTACCTCTGGG-3′ and 5′-AGCCTCCTCATCTTCCTGGT-3′ for human *MYO15A* c.3971C>A and c.4011insA mutations and 5′-TCAGAGGATTGTGCGCCTTT-3′ and 5′-ATGCTCAGTCTTCCTGGCAC-3′ for human *MYO15A* c.9690+1G>A mutation (BGI Inc., China). The PCR and amplification were performed according to a previous protocol [15]. Sequence alignment of *MYO15A* was performed using the DNASTAR software.

### 2.6. Functional Prediction

Next, phylogenetic analysis of different sequence alignments was performed by BioEdit. The included sequences were NP_057323.3 (human), XP_001077498.1 (rat), XP_015149897.1 (chicken), XP_009430930.1 (chimpanzee), XP_014974242.1 (monkey), XP_015323568.1 (cattle), and XP_015293218.1 (macaque).

### 2.7. Structure Modeling

Lastly, three-dimensional (3D) structure of the mutant and wild-type motor domain of *MYO15A* (NP_057323.3) was constructed by I-TASSER. These 3D models were visualized by Swiss-Pdb Viewer 4.01.

## 3. Result

### 3.1. Subjects and Clinical Findings

**Figure 1:**
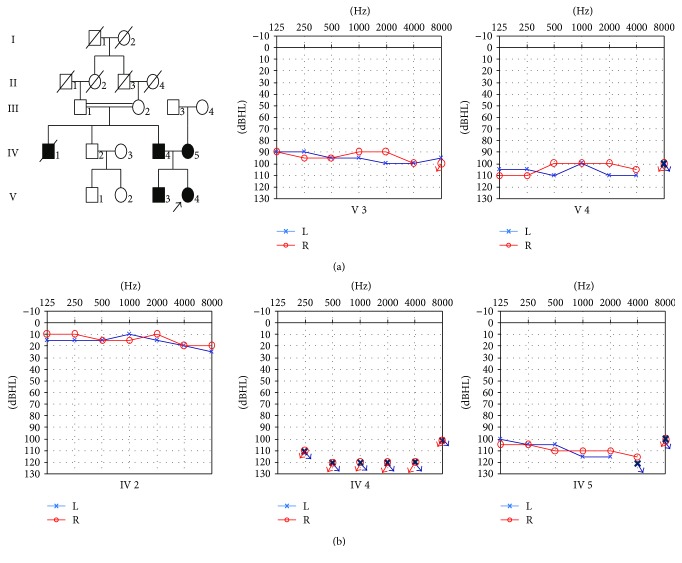
Clinical phenotype presentations of the pedigree. (a) Disease presentation of the family members and the pedigree map. All living members participated in the study, and V-3, V-4, and IV-2 were sequenced using capture gene sequencing. (b) Pure-tone audiograms of the family. Frequency in hertz (Hz) is plotted on the *x*-axis and the hearing level in decibels (dBHL) on the *y*-axis. The arrows in blue or red mean that the pure tones are not elicited at this point.

Four affected patients (V-3, V-4, IV-4, and IV-5) and eight unaffected members (V-1, V-2, IV-2, IV-3, III-1, III-2, III-3, and III-4) in the consanguineous Chinese family with ARNSHL participated in this study (Figure 1(a)). The proband was 17 years old and suffered from congenital deafness. As shown in Figure 1(b), all patients were affected with bilateral profound deafness with a flat audiogram revealed by PTA.

Clinical evaluations and otoscopy tests showed no abnormalities. Type A tympanograms were obtained from acoustic immittance tests, and there was no inner ear anomaly in patients revealed by MRI. ABR test revealed that the hearing loss of patients were all above 90 dBHL, and DPOAE were absent bilaterally from all patients. The clinical features of this family were summarized in Table 1.

### 3.2. Targeted Deafness Gene Capture Sequencing

All 127 hearing loss-related genes were sequenced in V-3, V-4, and IV-2 family members. Three mutations, c.3971C>A (p.A1324D), c.4011insA (p.Q1337Qfs^∗^22), and c.9690+1G>A, were detected in *MYO15A* gene (NM_016239), which were cosegregated with the disease, suggesting that these mutations may be the etiology of deafness in this ARNSHL family.

### 3.3. Confirmation of the Three Variants

**Figure 2:**
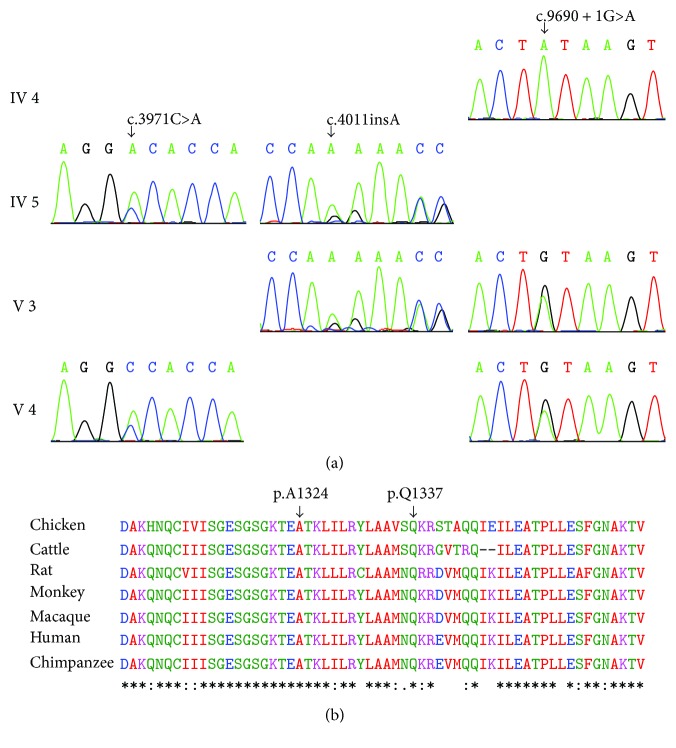
Sequence chromatograms and protein alignment. (a) The sequences of *MYO15A* at 3971, 4011, and 9690 + 1 of four family members. (b) The conservation of the p.A1324 and p.Q1337 residues (shown with black arrows) in healthy human, rat, chicken, chimpanzee, monkey, cattle, and macaque.

The three *MYO15A* variants were confirmed by direct sequencing in all participating persons. The homozygous *MYO15A* mutation c.9690+1G>A was detected in the affected patient IV-4. The compound heterozygous *MYO15A* variants c.3971C>A and c.4011insA were detected in the affected patient IV-5. The compound heterozygous c.3971C>A & c.9690+1G>A and c.4011insA & c.9690+1G>A were identified in the affected siblings, respectively (V-3, V-4) (Figure 2(a)). The other unaffected family members either carried only one heterozygous mutation or had a wild-type genotype (Table 2). However, these variants were absent in two hundred ethnically matched control persons. To our knowledge, c.3971C>A and c.4011insA variants were all considered as the first report and that three mutations identified in one family was quite rare.

The mutation c.3971C>A of *MYO15A*, a missense mutation, leads to an alternation of an alanine with an aspartic at amino acid position 1324 (p.A1324D). The protein structure change caused by this mutation is predicted next. The mutation c.4011insA is an insertion mutation predicting to lead to a reading frame shift at position 1337 and a stop codon (p.Q1337Qfs^∗^22) and truncate the translation of mRNA resulting in lack of its complete length. The mutation c.9690+1G>A was a splicing-site mutation resulting in a G to A transition at the donor splice site of intron 59. Thus, this mutation impaired the normal processing of mRNA formation.

The alignment of *MYO15A* from different species of human, rat, chicken, chimpanzee, monkey, cattle, and macaque was analyzed (Figure 2(b)). The result proved that p.A1324 and p.Q1337 were conservative among multiple species, which powerfully suggesting that these residues are important for the proper protein function.

### 3.4. Structural Modeling of p.A1324D in the Motor Domain of *MYO15A*

**Figure 3:**
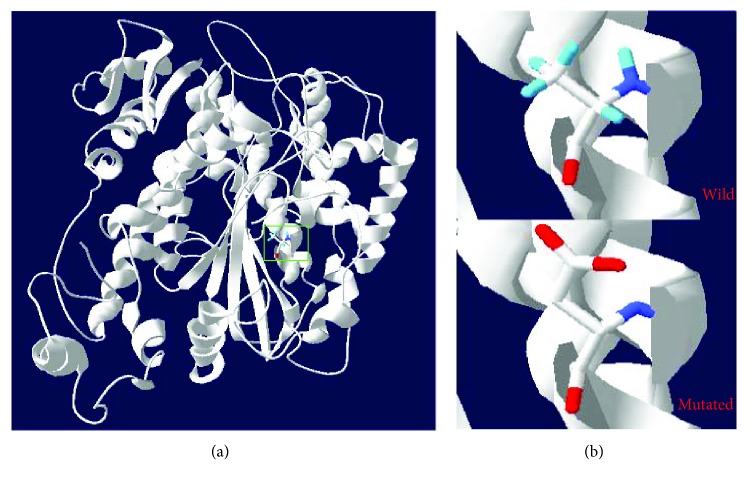
Structure modeling of wild-type and mutated MYO15A motor domain. (a) Green box shows the complete MYO15A motor domain. (b) The detailed structure of wild and mutated p.A1324 residue.

A 3D simulative structure (PDB ID:1g8xA) of *MYO15A* motor domain was built, which contained the myosin XVA protein residues 1261–1887. The sequence identity between the template and target was 44% which was higher than the average 25%. We used Swiss-Pdb Viewer 4.1 software to analyze the mutated and wild structures of motor domain (Figure 3). The variant was speculated to affect the side chain because of the missense change. The prediction uncovered that this mutation caused damage to the protein function by means of the change of protein structure.

## 4. Discussion

Changes in the *MYO15A* gene are known to be responsible for DFNB3 deafness. DFNB3 is the third pathogenic locus responsible for severe to profound ARNSHL. This report performed targeted capture sequence of 127 known deafness genes to describe genetic causes of a Chinese ARNSHL family. We identified three disease-causing *MYO15A* mutations finally. Multiple novel gene mutations revealed in one NSHL family are quite rare in the previous studies.

Our finding revealed that the variants p.A1324D and p.Q1337Qfs^∗^22 are located at myosin XVA motor domain. This region containing actin- and ATP-binding sites can produce the force that transports actin filaments in vitro [21]. The mutation p.A1324D is a missense change leading to an amino acid substitution, and p.Q1337Qfs^∗^22 results in a frameshift insertion and introduces a stop codon in the *MYO15A* open reading frame. A truncated protein is generated caused by the frameshift mutation. We estimate that the truncated protein is likely to be misexpressed or mislocated in the inner ear. Similarly to human DFNB3 hearing loss, a mouse model with homozygous variant in the myosin XVA motor domain exhibits profound hearing loss [22]. Many mutations were reported in this domain [8, 23] and were proposed probably to have deleterious effects on protein function.

Another mutation c.9690+1G>A was first reported in 2015 [24]. It is a splicing-site mutation resulting in a G to A transition at the donor site and is predicted to locate between the junctional region of the posterior MyTH4 domain and the FERM domain in myosin XVA. Similarly to the myosin VIIA MyTH4-FERM domain, this region is important for the localization to the stereocilia tips [25, 26], which is essential for the normal function of the stereocilia tips. Therefore, we speculate that this mutation could weaken the MyTH4-FERM interface that leads to the disease.

**Figure 4:**
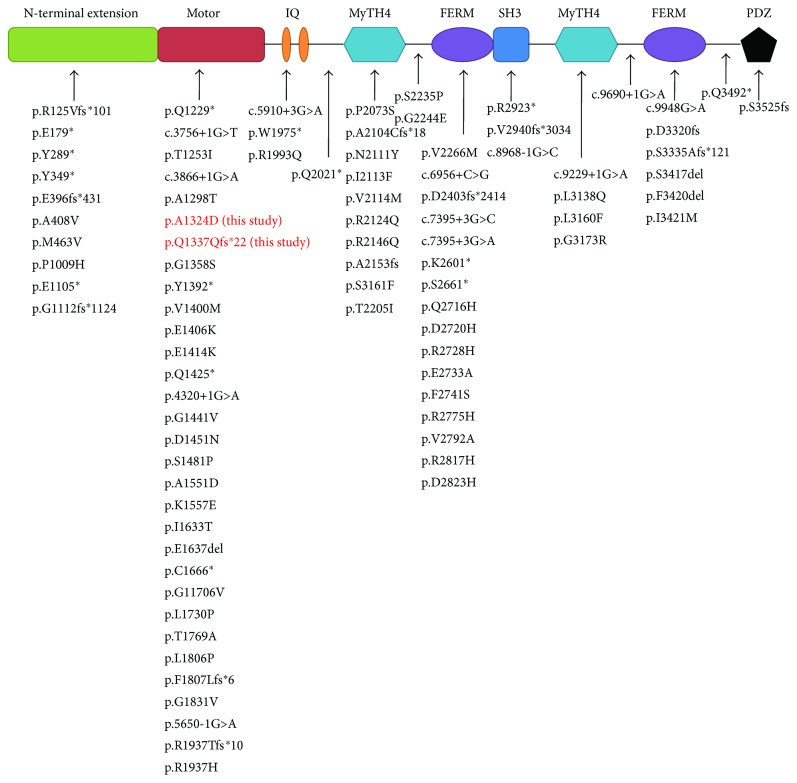
Reported mutations of *MYO15A* gene and their locations in the protein structure. The red characters refer to novel mutations identified in this study.

To date, a number of mutations in *MYO15A* gene have been identified in hereditary hearing loss (Figure 4). Interestingly, the mutation spectrum of *MYO15A* varies among different ethnic populations. For example, a relatively low frequency of 0.89% (10/1120) was reported in a study of Japanese deafness cohort [27]. A mutational frequency of 3.3% was revealed in the Middle and South Asian areas [28]. In another genetic analysis study, 4% (5/125) was found in a Chinese ethnic population preexcluding common mutations [29]. Furthermore, a frequency of 5% was found in a Pakistani recessively inherited severe to profound hearing loss population [30]. And a higher frequency of 5.71% was reported in an Iranian population preexcluding GJB2 mutations [31]. Therefore, for every country, consummating its own *MYO15A* mutation spectrum is essential for the diagnosis and prevention of hearing loss.

In this family, we identified three novel mutations in *MYO15A* gene causing profound hearing loss. Exactly as literatures state, *MYO15A* variants have usually been regarded as the cause of congenital severe to profound deafness [6, 7]. However, recently, some families suffering from less severe hearing impairment were also diagnosed a genetic cause of *MYO15A* mutations. We find those mutations leading to less severe hearing loss are usually located in the N-terminal domain in reported literatures. We estimate that the defect caused by the mutations in the N-terminal domain other than the rest of the domains might be compensated by the existence of class 2 isoform which has no N-terminal domain. The N-terminal extension domain plays a less important role than the other parts of the gene in the protein function. Chang et al.'s study in 2015 took the same ground [8].

In conclusion, three variants in *MYO15A* were identified using SNPscan assay and targeted capture sequencing: c.3971C>A (p.A1324D), c.4011insA (p.Q1337Qfs^∗^22), and c.9690+1G>A, and the first two of them were novel. All these mutations were cosegregated with the severe to profound deafness and were predicted to be pathogenic mutations. The present results also demonstrated that the combined application of SNPscan assay and targeted capture sequencing is a valuable and cost-saving molecular diagnostic strategy for ARNSHL. Our findings further extended the pathogenic variants of *MYO15A* gene in the ARNSHL group and would have a positive implication in genetic counseling for hearing loss families.

## Figures and Tables

**Table 1:** Clinical features of the family.

Subjects	Age	Age onset	Hearing loss	ABR	DPOAE	AR	MRI
IV-2	57 yr	−	Normal	−	−	−	Normal
IV-4	50 yr	0	Profound	95 dB	+	+	Normal
IV-5	47 yr	0	Profound	95 dB	+	+	Normal
V-3	19 yr	0	Profound	90 dB	+	+	Normal
V-4	17 yr	0	Profound	90 dB	+	+	Normal

+: presence; −: absence.

**Table 2:** Genotypes of all family members.

Family member	Genotype		Zygosity
	Variant 1	Variant 2	
III-1	c.9690+1G>A		Heterozygous
III-2	c.9690+1G>A		Heterozygous
III-3	c.4011insA		Heterozygous
III-4	c.3971C>A		Heterozygous
IV-2	c.9690+1G>A		Heterozygous
IV-3			Wild
IV-4	c.9690+1G>A		Homozygous
IV-5	c.3971C>A	c.4011insA	Compound heterozygous
V-1	c.9690+1G>A		Heterozygous
V-2			Wild
V-3	c.3971C>A	c.9690+1G>A	Compound heterozygous
V-4	c.4011insA	c.9690+1G>A	Compound heterozygous

## Supplementary Materials

Supplementary Materials Table S1: summary of the 127 targeted deafness genes.

## References

[1] Yan D., Tekin M., Blanton S., Liu X. Z. (2013). Next-generation sequencing in genetic hearing loss. *Genetic Testing and Molecular Biomarkers*.

[2] Cengiz F., Duman D., Sirmaci A. (2010). Recurrent and private *MYO15A* mutations are associated with deafness in the Turkish population. *Genetic Testing and Molecular Biomarkers*.

[3] Xia H., Huang X., Guo Y. (2015). Identification of a novel *MYO15A* mutation in a Chinese family with autosomal recessive nonsyndromic hearing loss. *PLoS One*.

[4] Kelsell D. P., Dunlop J., Stevens H. P. (1997). Connexin 26 mutations in hereditary non-syndromic sensorineural deafness. *Nature*.

[5] Wang A., Liang Y., Fridell R. (1998). Association of unconventional myosin *MYO15* mutations with human nonsyndromic deafness *DFNB3*. *Science*.

[6] Brownstein Z., Friedman L., Shahin H. (2011). Targeted genomic capture and massively parallel sequencing to identify genes for hereditary hearing loss in Middle Eastern families. *Genome Biology*.

[7] Gao X., Zhu Q., Song Y. (2013). Novel compound heterozygous mutations in the *MYO15A* gene in autosomal recessive hearing loss identified by whole-exome sequencing. *Journal of Translational Medicine*.

[8] Chang M., Kim A., Kim N. K. (2015). Identification and clinical implications of novel *MYO15A* mutations in a non-consanguineous Korean family by targeted exome sequencing. *Molecules and Cells*.

[9] Liang Y., Wang A., Belyantseva I. (1999). Characterization of the human and mouse unconventional myosin XV genes responsible for hereditary deafness *DFNB3* and shaker 2. *Genomics*.

[10] Berg J., Powell B., Cheney R. (2001). A millennial myosin census. *Molecular Biology of the Cell*.

[11] Garcia-Alvarez B., de Pereda J., Calderwood D. (2003). Structural determinants of integrin recognition by talin. *Molecules and Cells*.

[12] Krendel M., Mooseker M. (2005). Myosins: tails (and heads) of functional diversity. *Physiology*.

[13] Belyantseva I., Boger E., Friedman T. (2003). Myosin XVa localizes to the tips of inner ear sensory cell stereocilia and is essential for staircase formation of the hair bundle. *Proceedings of the National Academy of Sciences of the United States of America*.

[14] Beyer L., Odeh H., Probst F. (2000). Hair cells in the inner ear of the pirouette and shaker 2 mutant mice. *Journal of Neurocytology*.

[15] Zhang F., Bai X., Xiao Y. (2016). Identification of a novel mutation in *SLC26A4* gene in a Chinese family with enlarged vestibular aqueduct syndrome. *International Journal of Pediatric Otorhinolaryngology*.

[16] Carioli J., Teixeira A. (2014). Use of hearing AIDS and functional capacity in middle-aged and elderly individuals. *International Archives of Otorhinolaryngology*.

[17] Zhang F., Xiao Y., Xu L. (2016). Mutation analysis of the common deafness genes in patients with nonsyndromic hearing loss in Linyi by SNPscan assay. *BioMed Research International*.

[18] Ma Y., Xiao Y., Bai X. (2016). *GJB2*, *SLC26A4*, and mitochondrial *DNA12S rRNA* hot-spots in 156 subjects with non-syndromic hearing loss in Tengzhou, China. *Acta Oto-Laryngologica*.

[19] Du W., Cheng J., Ding H., Jiang Z., Guo Y., Yuan H. (2014). A rapid method for simultaneous multi-gene mutation screening in children with nonsyndromic hearing loss. *Genomics*.

[20] Wei X., Ju X., Yi X. (2011). Identification of sequence variants in genetic disease-causing genes using targeted next-generation sequencing. *PLoS One*.

[21] Redowicz M. (1999). Myosins and deafness. *Journal of Muscle Research & Cell Motility*.

[22] Probst F., Fridell R., Raphael Y. (1998). Correction of deafness in shaker-2 mice by an unconventional myosin in a BAC transgene. *Science*.

[23] Woo H., Park H., Baek J. (2013). Whole-exome sequencing identifies *MYO15A* mutations as a cause of autosomal recessive nonsyndromic hearing loss in Korean families. *BMC Medical Genetics*.

[24] Chen Y., Wang Z., Wang Z. (2015). Targeted next-generation sequencing in Uyghur families with non-syndromic sensorineural hearing loss. *PLoS One*.

[25] Wu L., Pan L., Wei Z., Zhang M. (2011). Structure of MyTH4-FERM domains in myosin VIIa tail bound to cargo. *Science*.

[26] Chai R., Li G., Wang J., Zou J. (2017). Hearing Loss: Reestablish the Neural Plasticity in Regenerated Spiral Ganglion Neurons and Sensory Hair Cells. *Neural Plasticity, article*.

[27] Miyagawa M., Nishio S., Hattori M. (2015). Mutations in the *MYO15A* gene are a significant cause of nonsyndromic hearing loss: massively parallel DNA sequencing-based analysis. *Annals of Otology Rhinology & Laryngology*.

[28] Nal N., Ahmed Z., Erkal E. (2007). Mutational spectrum of *MYO15A*: the large N-terminal extension of myosin XVA is required for hearing. *Human Mutation*.

[29] Yang T., Wei X., Chai Y., Li L., Wu H. (2013). Genetic etiology study of the non-syndromic deafness in Chinese Hans by targeted next-generation sequencing. *Orphanet Journal of Rare Diseases*.

[30] Bashir R., Fatima A., Naz S. (2012). Prioritized sequencing of the second exon of *MYO15A* reveals a new mutation segregating in a Pakistani family with moderate to severe hearing loss. *European Journal of Medical Genetics*.

[31] Fattahi Z., Shearer A., Babanejad M. (2012). Screening for *MYO15A* gene mutations in autosomal recessive nonsyndromic, *GJB2* negative Iranian deaf population. *American Journal of Medical Genetics Part A*.

